# Reconceptualizing the chlamydial inclusion as a pathogen-specified parasitic organelle: an expanded role for Inc proteins

**DOI:** 10.3389/fcimb.2014.00157

**Published:** 2014-10-31

**Authors:** Elizabeth R. Moore, Scot P. Ouellette

**Affiliations:** Division of Basic Biomedical Sciences, Sanford School of Medicine, University of South DakotaVermillion, SD, USA

**Keywords:** Chlamydia, pathogen specified organelle, inclusion membrane, inc protein, parasitophorous vacuole

## Abstract

*Chlamydia* is an obligate intracellular pathogen that develops in the host cell in a vacuole termed the chlamydial inclusion. The prevailing concept of the chlamydial inclusion is of a parasitophorous vacuole. Here, the inclusion is the recipient of one-way host-pathogen interactions thus draining nutrients from the cell and negatively impacting it. While *Chlamydia* orchestrates some aspects of cell function, recent data indicate host cells remain healthy up until, and even after, chlamydial egress. Thus, while *Chlamydia* relies on the host cell for necessary metabolites, the overall function of the host cell, during chlamydial growth and development, is not grossly disturbed. This is consistent with the obligate intracellular organism's interest to maintain viability of its host. To this end, *Chlamydia* expresses inclusion membrane proteins, Incs, which serve as molecular markers for the inclusion membrane. Incs also contribute to the physical structure of the inclusion membrane and facilitate host-pathogen interactions across it. Given the function of Incs and the dynamic interactions that occur at the inclusion membrane, we propose that the inclusion behaves similarly to an organelle-albeit one that benefits the pathogen. We present the hypothesis that the chlamydial inclusion acts as a pathogen-specified parasitic organelle. This representation integrates the inclusion within existing subcellular trafficking pathways to divert a subset of host-derived metabolites thus maintaining host cell homeostasis. We review the known interactions of the chlamydial inclusion with the host cell and discuss the role of Inc proteins in the context of this model and how this perspective can impact the study of these proteins. Lessons learnt from the chlamydial pathogen-specified parasitic organelle can be applied to other intracellular pathogens. This will increase our understanding of how intracellular pathogens engage the host cell to establish their unique developmental niches.

## Introduction

Bacteria that seek refuge from host defenses within a cell encounter a hostile intracellular environment. Toll-like receptors and other pattern recognition receptors (PRRs) recognize pathogen-associated molecular pattern molecules (PAMPs) such as lipopolysaccharide and peptidoglycan. Recognition of PAMPs leads to the activation of various signaling pathways that typically lead to either cell death via apoptosis or engulfment of the “foreign” bodies via autophagy with subsequent fusion of the vacuole with lysosomes. To this end, intracellular bacteria have devised various strategies to avoid or subvert these innate immune responses.

Most intracellular bacteria have intrinsic mechanisms for entering host cells, often through actin-mediated processes that ultimately lead to encapsulation of the invading bacterium within a membrane-bound compartment. Some bacteria, for example *Rickettsia* and *Listeria*, lyse this compartment and reside within the cytosol whereas others, including *Mycobacterium*, *Legionella*, *Salmonella*, *Coxiella*, *Anaplasma*, and *Chlamydia*, reside within it. For the latter type, these vacuoles are specifically suited to providing their resident bacteria an ideal environment to promote growth and development, but the vacuole must necessarily be remodeled by the bacterium to serve these purposes and to avoid the innate immune responses of the cell.

Not surprisingly, these parasitophorous vacuoles are typically envisioned as an isolated compartment acting to subvert host cell processes and to favor pathogen growth by grossly imbalancing cellular functions. However, if a bacterium favors intracellular growth over growth in other environments, then it is more reasonable to expect that the relationship between such a bacterium and its host may be less destructive than commonly viewed. In this review, we use an obligate intracellular pathogen, *Chlamydia*, to illustrate this by showing how a parasitophorous vacuole may be better envisioned as a pathogen-specified parasitic organelle.

## Chlamydial epidemiology

*Chlamydia trachomatis* is the chlamydial organism most commonly associated with human disease and one of the most common human pathogens. *C. trachomatis* serovars cause either blinding trachoma (Schachter, 1999) or the most common bacterial sexually transmitted disease (STD) (Datta et al., 2007). Primary chlamydial infections in women are often innocuous, which increases the likelihood that they will go untreated. This in turn leads to the ascension of *Chlamydia* from the cervix into the upper genital tract and the development of pelvic inflammatory disease, ectopic pregnancy, and/or infertility. The pervasiveness of this problem is illustrated by CDC surveillance numbers, which estimate that 10% of women between the ages of 15 and 19 test positive for *Chlamydia* (Prevention, 2013). In the US, the CDC estimates that the cost of chlamydial genital infections exceeds 700 million dollars annually (Satterwhite et al., 2011). A hallmark of chlamydial infection is the ability of the pathogen to thrive within the host while limiting host immunological responses and obvious signs of inflammation (Darville and Hiltke, 2010). In fact, 80% of women who have a single incidence of a chlamydial infection do not develop clinical complications (Paavonen and Eggert-Kruse, 1999; Van Valkengoed et al., 2004), which is consistent with the initial chlamydial infection not being overtly detrimental to the host. However, chronic or recurrent chlamydial infections are quite common, especially in women, increasing the risk of ascending genitourinary infections and damage to the oviducts (Burstein et al., 1998; Molano et al., 2005; Darville and Hiltke, 2010). These data support the “cellular paradigm of chlamydial pathogenesis,” which states that, upon infection, non-immune cells mount a cytokine response which is both necessary *and* sufficient to exact tissue damage (Stephens, 2003). With chronic or recurring infections, this cytokine response will be sustained with increasing likelihood that *Chlamydia* will ascend within the female genital tract. Current prevention and treatment strategies fail to reduce the incidence of new infections and subsequent sequelae.

## The chlamydial developmental cycle

Chlamydia are obligate intracellular bacterial pathogens that utilize a developmental cycle to alternate between infectious, metabolically quiescent elementary bodies (EBs) and non-infectious, metabolically-active reticulate bodies (RBs) during a productive growth cycle (see Abdelrahman and Belland, 2005 for review). The two forms of *Chlamydia* reflect their distinct roles within the developmental cycle: the EB mediates attachment and internalization into a susceptible host cell and the RB grows and divides similarly to other bacteria. The developmental cycle occurs within a vacuole termed the inclusion. The molecular events required for differentiation between these morphologic forms are not fully understood. However, genome-wide microarray and targeted transcriptional studies have helped define genes that may be important at critical stages such as the EB-to-RB (early) and RB-to-EB (late) transition (Shaw et al., 2000; Belland et al., 2003; Nicholson et al., 2003).

The normal progression of chlamydia through the developmental cycle can be blocked by host immune effectors, β-lactam antibiotic treatment, and nutrient deprivation (Beatty et al., 1993, 1994). This leads to the establishment of persistent forms of the organism that are impaired in division but remain viable (Beatty et al., 1993). These persistent forms may be connected to the development of chronic infections and the sequelae with which they are associated (Thejls et al., 1991; Campbell et al., 1993; Bragina et al., 2001; Hjelholt et al., 2011). Reactivation of chlamydial growth and completion of the developmental cycle occurs upon removal of the stress (Beatty et al., 1994, 1995). How *Chlamydia* maintain the integrity of the inclusion during persistent growth is not well-understood.

As an obligate intracellular pathogen, *Chlamydia* must necessarily engage host cell membranes. It is well-characterized that the EB interacts and locally modifies the plasma membrane to promote endocytosis (Zeichner, 1982; Wyrick et al., 1989; Zhang and Stephens, 1992; Carabeo and Hackstadt, 2001; Carabeo et al., 2002, 2004; Davis et al., 2002; Conant and Stephens, 2007). For the RB, it is the inclusion membrane (IM) that serves as the means by which the bacterium communicates with the host cell (Ward, 1988). It is well-established that chlamydial protein synthesis and type III secretion are necessary for the early remodeling of the plasma membrane-derived IM (Fields et al., 2003; Scidmore et al., 2003). Chlamydial proteins that are type III secreted and remodel the IM are called Incs (Subtil et al., 2001; Dehoux et al., 2011).

## The inclusion evolves during the chlamydial developmental cycle

Inherent to the survival of all chlamydial species is the avoidance of the inclusion from the lysosomal pathway and other innate immune defenses. Hence, specific interactions with the host cell are orchestrated by the pathogen via the IM. Not surprisingly, this coincides with shifting nutrient sources as the organism positions itself to obtain nutrients without triggering a stress response from the host cell. For example, Ouellette et al. demonstrated that *Chlamydia* is dependent on lysosomal degradation products earlier in its developmental cycle whereas it preferentially utilizes free amino acids later in the cycle (Ouellette et al., 2011). Further, species and strains that grow faster are less dependent on these lysosomal degradation products as they more effectively utilize free amino acids from the host cell (Ouellette et al., 2011). Similarly, lipids may be supplied through direct recruitment of host cell enzymes early in the cycle [e.g., CERT (Derre et al., 2011; Elwell et al., 2011)] and vesicular-derived sources later in the cycle [e.g., via interactions with SNAREs (Kabeiseman et al., 2013)]. One likely result of these changes in nutrient availability/competition is that the IM is continually modified by Incs that are expressed at different stages of the chlamydial developmental cycle. We propose that, by altering the protein content of the IM and shifting acquisition of nutrient pools, *Chlamydia* is engaging a strategy to limit gross stress to the host cell. To accomplish this, the inclusion must be integrated within the host cell as opposed to existing in an isolated compartment that is unresponsive to changes in the host cell.

## Chlamydia is a highly evolved pathogen

In adapting to the obligate intracellular lifestyle, *Chlamydia* has significantly reduced its genome size as it relies on the host cell for most of its metabolic needs (McClarty, 2004). For example, *C. trachomatis* encodes 895 open reading frames (ORFs) in 1.04 Mbp (1.16 kbp/ORF) with little evidence of pseudogenes (Stephens et al., 1998). For comparison, *Rickettsia prowazekii*, another obligate intracellular pathogen, encodes 835 ORFs in 1.11 Mbp (1.33 kbp/ORF) yet contains many pseudogenes (Andersson et al., 1998). This indicates that *R. prowazekii* is still undergoing a reductive evolutionary process as it adapts to obligate intracellular life in its host and reduces its genome size. *Chlamydia*, meanwhile, is already highly adapted to this niche. These genomic data, along with the observation that *Chlamydia* transcribes most, if not all, genes, suggest that every ORF is important to *Chlamydia* (Belland et al., 2003; Nicholson et al., 2003).

## The chlamydial inclusion and Inc proteins

Early studies attempting to identify components of the inclusion revealed the presence of chlamydial proteins inserted into the IM (Bannantine et al., 1998; Scidmore-Carlson et al., 1999). These proteins are referred to as Inc proteins. Incs have one key, identifying motif: a large hydrophobic region encoding two transmembrane domains. With the publication of the first chlamydial genome and using this key characteristic, bioinformatics studies have estimated that *C. trachomatis* encodes greater than 50 *inc* genes (Lutter et al., 2012). This represents approximately 6% of the coding capacity of the organism (Stephens et al., 1998). Given the genome reduction previously described, this indicates that the Incs serve an important function. Further, many Incs are expressed at distinct times during the developmental cycle suggesting temporally orchestrated functions (Shaw et al., 2000; Belland et al., 2003; Nicholson et al., 2003). Other studies have shown that Incs encode a type III secretion signal that allows the bacterium to secrete these proteins to be subsequently inserted into the IM (Fields et al., 2003). Type III secretion systems are virulence factors of Gram-negative pathogens (Hueck, 1998) and, in *Chlamydia*, are linked to chlamydial survival and virulence (Betts-Hampikian and Fields, 2010). Because of this, Incs are likely virulence proteins, playing a key role in chlamydial growth and development. Topological analyses indicate that both the N- and C-termini of Incs are exposed on the cytoplasmic side of the IM, and a recent study, using an anti-FLAG antibody, showed that the C-terminus of IncD-FLAG is indeed exposed to the cytoplasm (Agaisse and Derre, 2014; Bauler and Hackstadt, 2014). In spite of the large number of Incs, only a few have been shown to interact with a host cell component (Scidmore and Hackstadt, 2001; Derre et al., 2011; Lutter et al., 2013).

The expression of Incs can be broadly divided into two temporal categories: those expressed early (i.e., soon after infection) and those that are expressed mid-cycle (i.e., once the inclusion is established). In these contexts and related to shifting nutritional needs, one would predict that early functioning Incs are essential for establishing the nascent inclusion and therefore essential to the organism. In contrast, later functioning Incs are dispensable, as they are predicted to impact the efficiency of nutrient acquisition thus affecting only the growth rate of the organism but not necessarily its ability to complete the developmental cycle. Indeed, only one Inc mutant has been characterized to date: IncA (Suchland et al., 2008). IncA is expressed mid-cycle and has been extensively studied and shown to contain two SNARE-like motifs that likely promote homodimeric interactions (Delevoye et al., 2008; Ronzone and Paumet, 2013). When host cells are infected with multiple EBs, IncA wild-type inclusions will fuse whereas mutant inclusions fail to fuse, thus IncA is not essential to chlamydial growth (Suchland et al., 2008). However, the IncA mutant did show a reduced rate of production of infectious progeny probably due to competition between the non-fused inclusions (Suchland et al., 2008). Overall, these observations suggest that *Chlamydia* modifies the IM to meet its evolving needs during the developmental cycle and that Incs are central to the function of the IM as a whole.

Although molecular mechanisms for inclusion biogenesis are lacking, many studies have described various characteristics of the inclusion. The nascent inclusion is rapidly diverted from the endocytic pathway after completion of the chlamydial-specified phagocytosis process and neither acquires markers such as Rab5 and EEA1 that identify early endocytic compartments nor markers such as Rab7 and LAMP1 that identify late endocytic compartments (Taraska et al., 1996; Scidmore et al., 1996b, 2003). The inclusion is trafficked to the microtubule organizing center where it selectively interacts with various host cell pathways (Grieshaber et al., 2003). For example, *Chlamydia* will intercept fluorescent, exocytically-derived, sphingomyelin (Hackstadt et al., 1996; Moore et al., 2008). More recent work by Moore et al. has shown that *Chlamydia* intercepts the exocytic SNAREs, syntaxin 6, and VAMP4 (Moore et al., 2011; Kabeiseman et al., 2013). For these reasons, the chlamydial inclusion is typically referred to as a parasitophorous vacuole that resides within a specialized exocytic compartment.

## The inclusion as a pathogen-specified parasitic organelle

**Figure 1:**
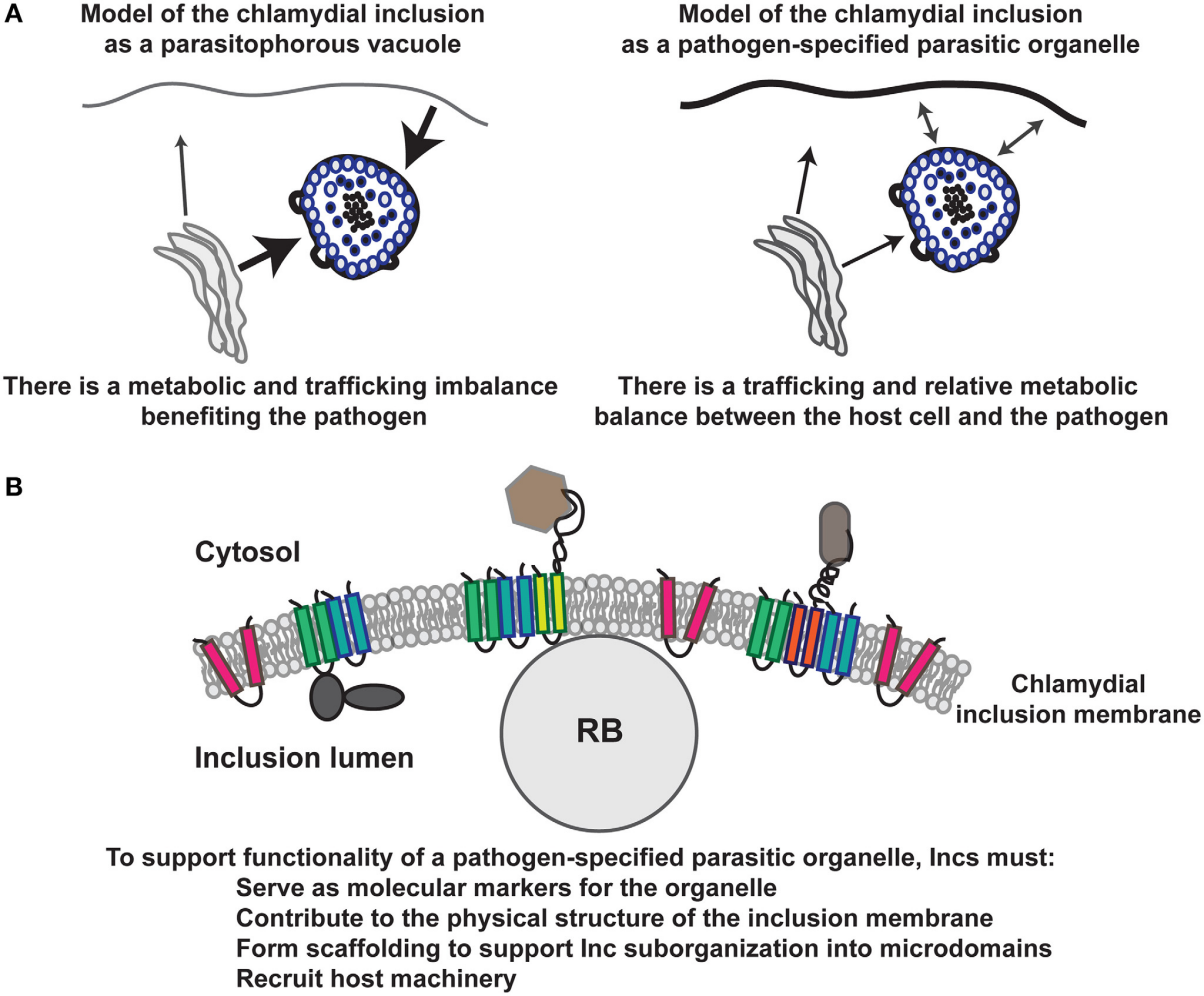
**Model of the function of the chlamydial inclusion**. In **(A)**, there are two representations of the chlamydial inclusion: the classical paradigm of the chlamydial inclusion as a parasitophorous vacuole and our concept of the chlamydial inclusion as a pathogen-specified parasitic organelle, which is more consistent with recent data. In **(B)**, the composition of the inclusion membrane is represented with Inc proteins serving as scaffolds to organize the membrane by creating microdomains to support host-chlamydial interactions and organize the inner leaflet of the chlamydial inclusion membrane. In our model, microdomains are collections of discrete subsets of Inc proteins as described in Mital et al. (2010) and Alzhanov et al. (2009).

An organelle is defined as a specialized subcompartment of a cell that serves a specific function. It is usually membrane-bound and is identified through various specific surface, cytoplasmically exposed markers. Proteins and/or vesicles destined for a particular organelle also contain a specific trafficking signal that allows the protein/vesicle to be targeted to the organelle. Given these characteristics of an organelle, we hypothesize that the chlamydial inclusion is a pathogen-specified parasitic organelle (Figure 1). In this context, the “chlamydial” organelle (i) serves the specific function of promoting chlamydial growth and development, (ii) is membrane-bound and identified through specific surface, cytoplasmically exposed markers: the Incs, and (iii) intercepts proteins/vesicles with specific trafficking signals (e.g., YGRL) (Moore et al., 2011; Kabeiseman et al., 2014). The inclusion-as-an-organelle model incorporates the dynamic characteristics of the inclusion that allow it to minimize its impact on host cell functions while promoting growth of the pathogen. The prevailing inclusion-as-a-parasitophorous-vacuole model is being challenged by current data in the field since it fails to account for the dynamic interactions that occur between this compartment and the host cell. A parasitophorous vacuole implies a one-sided relationship with the host cell that is detrimental to the host. Importantly, *Chlamydia*-infected cells are generally healthy until the end of the cycle when the EBs are ultimately released from the host cell. Nevertheless, even this process does not always result in cell death (Beatty, 2007; Hybiske and Stephens, 2007).

The inclusion clearly interacts, in a selective manner, with the exocytic pathway (Hackstadt et al., 1996; Scidmore et al., 1996a; Moore et al., 2008). Yet, it has also been shown to interact with transferrin receptor, late recycling endosomes, and other compartments, like multivesicular bodies (Rzomp et al., 2003, 2006; Beatty, 2006; Ouellette and Carabeo, 2010; Ouellette et al., 2011). Importantly, trafficking of glycoproteins to the cell surface of chlamydial infected cells is unimpeded (Scidmore et al., 1996a; Taraska et al., 1996; Ouellette and Carabeo, 2010), indicating that *Chlamydia* minimizes its impact on exocytic events. Similarly, only a small proportion (approximately 10%) of transferrin and transferrin receptor traffics to the inclusion, via the slow recycling pathway (Ouellette and Carabeo, 2010), and transferrin receptor recycling is not generally altered in chlamydial infected cells (Scidmore et al., 1996a; Taraska et al., 1996; Vanooij et al., 1997; Ouellette and Carabeo, 2010). Rab GTPases, members of the Ras superfamily of monomeric, low molecular weight G proteins, are well-established markers of specific subcellular organelles (Stenmark, 2009; Jean and Kiger, 2012). The chlamydial inclusion formed by *C. trachomatis*, for example, recruits Rabs 1, 4, 6, 11, and 14 (Rzomp et al., 2003, 2006; Capmany and Damiani, 2010), indicating that the inclusion interfaces with ER to Golgi transport pathways (Rab 1) (Allan et al., 2000; Moyer et al., 2001; Stenmark, 2009), recycling endosomes (Rabs 4 and 11) (McCaffrey et al., 2001; Zhang et al., 2004), Golgi and trans-Golgi networks (Rabs 6 and 14) (Goud et al., 1990; Antony et al., 1992; Junutula et al., 2004), and the cytokinetic apparatus (Rab 11) (Hehnly et al., 2012). More recent work from the Moore lab suggests that *Chlamydia* may intercept SNAREs trafficked *from* the plasma membrane rather than those being delivered *to* it (Kabeiseman et al., 2014). Further studies have shown that “membrane contact sites” form between the chlamydial IM and the endoplasmic reticulum, likely through IncD (Derre et al., 2011). Combined, these data indicate that the ultimate composition and identity of the chlamydial inclusion rely on its ability to interact with multiple subcellular pathways. This is consistent with “fitting in” within a network of organelles that engage in cross-talk to effectively orchestrate host cell functionality.

**Figure 2:**
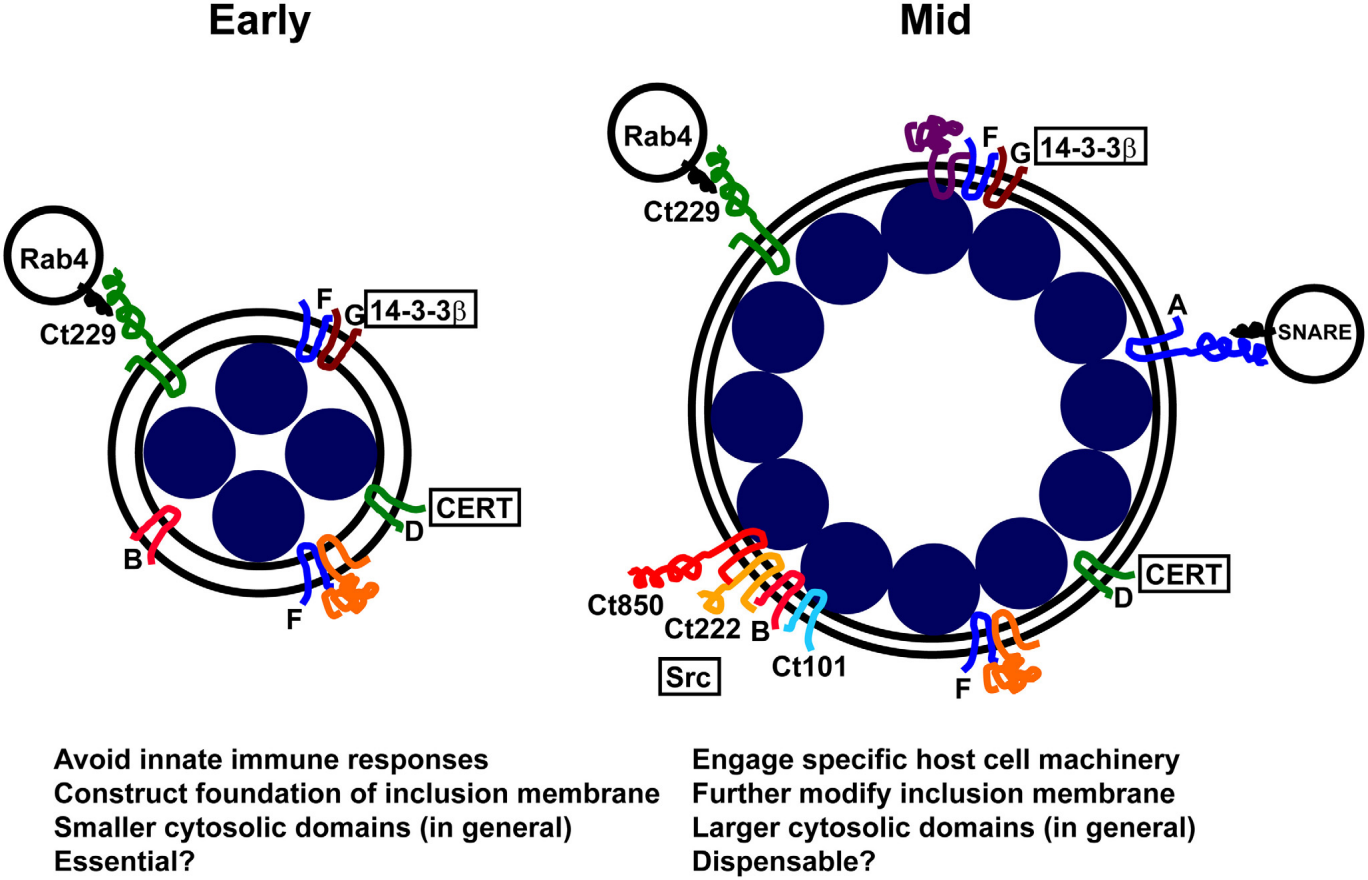
**Hypothetical model for temporal changes in the inclusion membrane with proposed functions**. Boxed and circled proteins are known host-cell derived proteins that localize to the inclusion. Unboxed proteins or letters represent Incs. The only interaction to be validated by multiple biochemical assays demonstrating in-cell interactions is IncD and CERT (Agaisse and Derre, 2014).

The view of the inclusion as an isolated parasitophorous vacuole also prevails when considering the function of Incs at the IM. Incs are commonly thought to function as mediators of host cell interactions by binding host cell components. Specifically, within the parasitophorous vacuole model, Incs sequester host cell components, effectively removing them from canonical trafficking pathways. Because of this prevailing model, little thought has been given to potential contributions of Incs to the structure and function of the IM itself. For example, what protein-protein or protein-lipid interactions are required to form this membrane? Hence, many Incs may function by organizing and providing structure to the IM. This is an especially important consideration given that a quarter of putative Inc proteins have very limited cytoplasmic domains (< 30 amino acids), coupled with the fact that only a limited number of Incs have been shown to interact *in vitro* with host cell proteins (Scidmore and Hackstadt, 2001; Delevoye et al., 2008; Derre et al., 2011; Lutter et al., 2013). Most of these encode coiled-coil domains that are commonly used motifs in protein-protein interactions thus increasing the likelihood of detecting potential interacting partners. Therefore, we hypothesize that Incs have one of two functions: (i) to construct the IM itself and promote interactions between Incs or (ii) to selectively interact with host cell components (Figure 2).

One likely function of the smaller Inc proteins is thus to serve as a scaffold within the IM to both give it structure and create microdomains to facilitate interactions between Inc proteins. *C. trachomatis* IncB, for instance, co-localizes with Ct101, Ct222, and Ct850 at later times during infection, and this complex is thought to recruit Src family kinases (Mital et al., 2010). Yet IncB must have another function given its early expression pattern and small size. This function may be to create microdomains, or collections of specific proteins to promote a specific function, within the IM to facilitate interactions between Incs as well as with host cell proteins. The view of the inclusion as a pathogen-specified parasitic organelle integrates the developmental expression of Incs with temporal characteristics of the inclusion and the bacteria themselves in ways that allow the inclusion to have a minimal impact on the health of the host cell.

## Limitations of studying the inclusion membrane

When studying an organelle, it can typically be purified from the host cell and, subsequently, membranes from this organellar fraction can be isolated to determine the protein and lipid composition of the organelle in question. In the case of the chlamydial inclusion, the membrane is highly fragile and typically falls apart upon cell lysis, regardless of the conditions. Further, because of the fragility of the inclusion, the IM is refractory to biochemical purification (Saka et al., 2011). Therefore, purification of chlamydial inclusions or IM fractions is not possible. Once the integrity of the IM is compromised, organisms freely associate with various organelles. In fact, when purifying chlamydial organisms from host cells, two density gradient steps are required to eliminate host cell debris from the purified organisms (Caldwell et al., 1981; Scidmore, 2005). The inability to purify intact chlamydial inclusions from host cells precludes any attempts at characterizing the proteome and interactome of this compartment. Hence, specific molecular events that occur and are responsible for IM composition and integrity are unknown.

Despite the dearth of knowledge surrounding the composition of the IM, 50 + Inc proteins have been characterized to reside in the IM (Bannantine et al., 1998; Scidmore-Carlson et al., 1999; Li et al., 2008), indicating that Inc proteins are good tools for studying the chlamydial IM. However, Inc proteins are membrane proteins, and membrane proteins are notoriously difficult to study due to their hydrophobicity. Prior studies searching for interacting partners of Incs have typically relied on expressing the cytosolic domains of a given Inc in yeast two-hybrid systems or purifying a recombinant version of the cytosolic domain for pull-downs and then ascertaining if the identified host binding partners co-localize to the chlamydial inclusion via indirect immunofluorescence (Scidmore and Hackstadt, 2001; Delevoye et al., 2008; Derre et al., 2011; Lutter et al., 2013). With regard to yeast two-hybrid systems, this method of testing interactions is lacking a contextual link to chlamydial biology (i.e., the Inc is expressed by eukaryotic machinery and must interact in the nucleus of the yeast cell with its binding partner). With regard to pull-downs, the cytosolic domains that have been studied contain coiled-coil domains (e.g., IncA, Ct228, Ct229), which are commonly used motifs in protein-protein interactions thus increasing the likelihood of a positive hit. A single publication exists examining expression of an Inc and recruitment of host proteins *in vivo* (Agaisse and Derre, 2014). Further, only a single publication exists that biochemically examines interactions between Incs (Mital et al., 2010).

## Is the pathogen-specified parasitic organelle unique to chlamydia?

Many intracellular pathogens, both obligate and facultative, establish a compartment in which they replicate. These are often referred to as a pathogen-containing vacuole (e.g., the SCV of *Salmonella* is the *Salmonella*-containing vacuole). Inevitably, the pathogen modifies the compartment in ways that ensure its growth and survival, and this is accomplished by the secretion of effectors into the host cell-whether the membrane of the compartment or the cytosol. The Gram-negative pathogens typically encode multiple secretion systems, such as the Type III secretion system similar to what is found in *Chlamydia* or the Type IV system used by *Legionella*. *Salmonella* type III effectors ensure the stabilization and maintenance of the SCV (Figueira and Holden, 2012). The obligate intracellular *Anaplasma phagocytophilum* recruits cholesterol to its intracellular vacuole and employs Type IV secretion to modulate autophagy (Lin and Rikihisa, 2003; Truchan et al., 2013). *Mycobacterium* also relies on secreted effectors to delay the maturation of its compartment along the endocytic pathway. *Mycobacterium tuberculosis* thrives within alveolar macrophage and dendritic cells. Key to its intracellular survival is the ability to prevent acidification of its phagosome (known as the MCV, *Mycobacterium* containing vacuole) to circumvent its destruction via autophagy (Sturgill-Koszycki et al., 1994). Carefully orchestrating the fate of the organelle includes secreting effectors such as SapM, which consumes phosphatidylinositol-3-phosphate from the host to alter endosomal maturation (Vergne et al., 2005). Additionally, the organism incorporates the lipid sulfatide into its cell wall to inhibit macrophage production of radical oxygen species or to increase phagocytosis (combined, a process known as “priming”) (Pabst et al., 1988). Additionally, sulfatide within the mycobacterial cell wall acts as a repellant to prevent fusion with lysosomal or autophagosomal compartments (Goren et al., 1976). From within the MCV, *M. tuberculosis* inhibits apoptosis as a direct means to avoid effectorcytosis, which is the clearance of apoptotic cells by uninfected macrophages (Keane et al., 2000). Hence, the ability of the MCV to function as an organelle depends on a very carefully, well-adapted choreography between host and microbe. Indeed, all of these examples are considering the pathogen as exploiting the host—as in the pathogen (the aggressor) is taking advantage of the host cell (the victim). However, the best way to sustain an infection is not to dominate or weaken the host but to achieve a plane of equilibrium. This equilibrium is achievable by coopting space and resources in the context of a pathogen-specified parasitic organelle.

One of the earliest examples of a pathogen-specified parasitic organelle was the observation that *Legionella pneumophila* creates an endoplasmic reticulum-derived niche within macrophages (Horwitz and Silverstein, 1980; Horwitz, 1983a,b). Considered an opportunistic pathogen, *L. pneumophila* coexists within amoeba in the environment and, when accidentally acquired by a human, creates a similar relationship with the amoeba-like macrophage. Critical to the identity of an organelle is the engagement of very specific trafficking machinery. For example, *L. pneumophila* recruits the low molecular weight GTPase Rab1 to the LCV or *Legionella* containing vacuole (Derre and Isberg, 2004; Kagan et al., 2004). It is well-established that Rab proteins are “markers” for distinct organelles (Stenmark, 2009; Jean and Kiger, 2012). For example, Rab1 is a marker for the ER (Allan et al., 2000; Moyer et al., 2001; Stenmark, 2009). To manipulate Rab1 function at the LCV, two Type IV effectors are produced: LepB, which switches Rab1 “off” by acting as a GTPase activating protein (GAP) (Ingmundson et al., 2007; Goody et al., 2012) and DrrA, which switches Rab1 “on” by acting as a guanine nucleotide exchange factor (GEF) (Machner and Isberg, 2006; Murata et al., 2006). By modulating Rab1 activity at the LCV, *Legionella* ultimately orchestrates the recruitment of specific syntaxin/SNARE machinery to the LCV to maintain an ER-like identity by promoting fusion of ER-derived vesicles with the LCV (Arasaki et al., 2012). Further, *Legionella* produces an effector, VipD, which binds to Rab5 and Rab22 specifically to prevent these Rabs from trafficking and recruiting endosomal membranes to the LCV (Ku et al., 2012).

## Conclusions and future directions

Clearly, the term “pathogen-specified parasitic organelle” is not a definition solely applicable to the chlamydial inclusion. From this perspective, rather than looking at families of effectors for commonalities between intracellular organisms, we can instead examine similar mechanisms of pathogen-specified parasitic organelle establishment. The exploitation of Rab proteins (as an example of host cell machinery) is an excellent example of this. While Rab proteins are specific for different subcellular compartments, a given Rab protein functions similarly at every location within the cell—even, it would seem, at pathogen-specified parasitic organelles.

**Figure 3:**
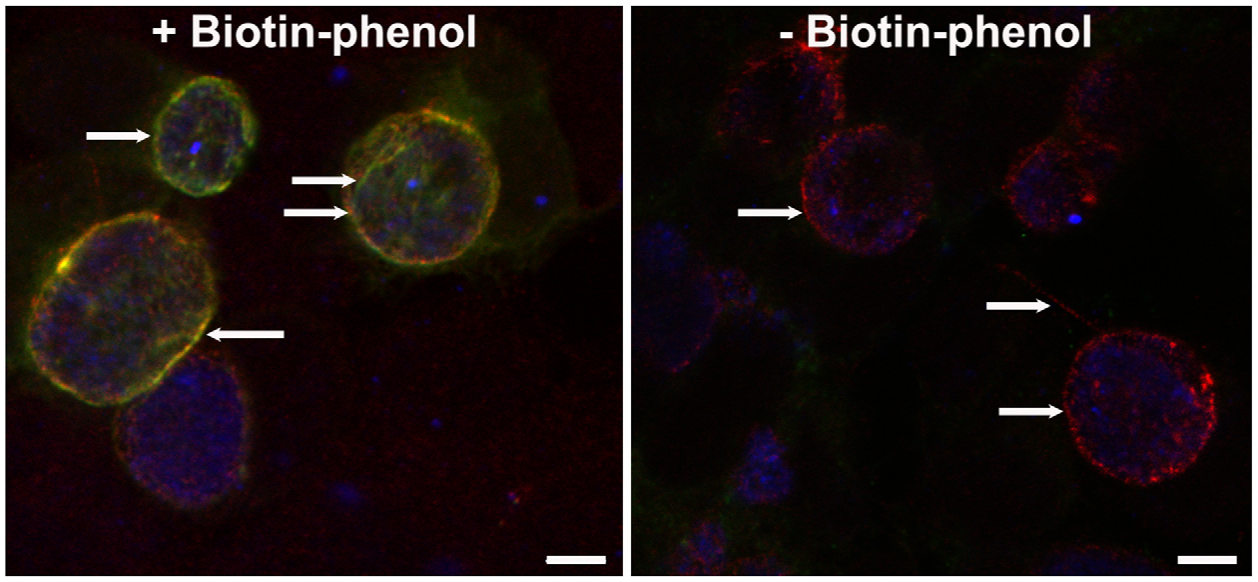
**Inducible expression of IncA_TM_-APEX2**. HeLa cells were inoculated with *C. trachomatis* serovar L2 transformed with pASK-IncA_TM__APEX2-mKate2::L2 to allow for inducible expression of the N-terminal region of IncA encoding the transmembrane domains (TM) fused to APEX2. The plasmid backbone (pASK-GFP/mKate2-L2 plasmid) for this study was generously provided by P. Scott Hefty (Department of Molecular Biosciences, University of Kansas) (Wickstrum et al., 2013). 6 h after infection, IncA_TM_-APEX2 expression was induced by treating cultures with 10 ng/ml anhydrotetracycline for an additional 18 h. Monolayers were then treated with or without biotin-phenol, processed for immunofluorescence and visualized with a 60X objective on an Olympus Fluoview 1000 Laser Scanning Confocal Microscope. White arrows depict IncA_TM_-APEX2 (red) localized to the IM or fibers extending from the inclusion. In the presence of biotin-phenol the construct is able to biotinylate (green, detected with streptavidin-488) the IM. Scalebar = 5 μm.

Additionally, some secreted bacterial proteins, such as Incs, may help to construct the membrane of pathogen-specified parasitic organelles. Historically, Inc proteins have been examined to determine with which host protein they interact as a means to subvert host cell function, not in how they interact with one another with regards to organization of the chlamydial inclusion. Both interactions are relevant in the context of how the inclusion functions within the host cell. New approaches in working with membrane proteins will need to be implemented to circumvent these difficulties. One approach is the bacterial adenylate cyclase-based two hybrid system (BACTH), which can examine *in vivo* protein-protein interactions within the context of a phospholipid bilayer (Karimova et al., 1998; Ouellette et al., 2014). Another approach is proximity labeling techniques to covalently modify neighboring proteins using either a promiscuous biotin ligase (Roux et al., 2012) or an ascorbate peroxidase (Rhee et al., 2013) fused to a protein of interest. In either case, expression of the tagged construct followed by exogenous addition of biotin (BirA^*^) or biotin-phenol (APEX) will result in interacting partners being tagged with biotin. Hence, protein-protein interactions are recorded and tagged within the cell prior to lysis. Therefore, measurement of protein-protein interactions is levied against the affinity of biotin for streptavidin in a pull-down and not for the preservation of these interactions during lysis and immunoprecipitation. In our hands, we have been able to transform *C. trachomatis* serovar L2 with an IncA-APEX construct, which biotinylates the IM upon addition of biotin-phenol (Figure 3). These methodologies will rapidly improve our ability to not only characterize interactions of membrane proteins but to map previously unpurifiable cellular organelles.

Granted, if we accept that the chlamydial inclusion is a pathogen-specified parasitic organelle, then we assume that the inclusion is interfacing with other subcellular compartments in an equitable manner. This, in turn, increases the complexity of studying these interactions. However, by combining biochemical protein-protein interactions with imaging technology, like photoconvertible protein constructs and TIRF (total internal reflection fluorescence microscopy; (Axelrod, 1981; Axelrod et al., 1983; Lin and Hoppe, 2013), we can demonstrate, for the first time, crosstalk between a pathogen-specified parasitic organelle and host cell organelles. Most likely, many of these transmembrane bacterial proteins share similarities in how they organize membranes within eukaryotic cells.

The ability to create unique antimicrobials depends upon us, the researchers, being able to adequately target survival mechanisms that are unique to pathogens without impacting the host. Further, a class of antimicrobials could be created that target specific pathogen-specified parasitic organelles that cordons off the pathogen in a manner that, rather than encouraging adaptation, limits destructive activities toward the host. The concept of “pathogen-specified parasitic organelles” can radicalize our thinking in how we examine intracellular pathogens and develop novel antimicrobial strategies against them.

### Conflict of interest statement

The authors declare that the research was conducted in the absence of any commercial or financial relationships that could be construed as a potential conflict of interest.
